# Impaired illness awareness in schizophrenia and posterior corpus callosal white matter tract integrity

**DOI:** 10.1038/s41537-019-0076-x

**Published:** 2019-04-29

**Authors:** Philip Gerretsen, Tarek K. Rajji, Parita Shah, Saba Shahab, Marcos Sanches, Ariel Graff-Guerrero, Mahesh Menon, Bruce G. Pollock, David C. Mamo, Benoit H. Mulsant, Aristotle N. Voineskos

**Affiliations:** 1 Campbell Family Mental Health Research Institute, Centre for Addiction and Mental Health, (CAMH), Toronto, Canada; 2 University of Toronto, Toronto, ON Canada; 3 Krembil Centre for Neuroinformatics – CAMH, Toronto, ON Canada; 4 Department of Psychiatry, University of British Columbia, Vancouver, BC Canada; 5 University of Malta, Msida, Malta

**Keywords:** Schizophrenia, Biomarkers, Neural circuits

## Abstract

Impaired illness awareness (Imp-IA) in schizophrenia is associated with interhemispheric imbalance, resulting in left hemisphere dominance, primarily within the posterior parietal area (PPA). This may represent an interhemispheric “disconnection syndrome” between PPAs. To test this hypothesis, we aimed to determine if diffusion-based measures of white matter integrity were disrupted in the corpus callosal tracts linking PPAs (i.e., splenium) in patients with Imp-IA in schizophrenia. T1-weighted and diffusion-weighted scans were acquired on a 1.5T GE scanner for 100 participants with a DSM-IV-TR diagnosis of schizophrenia and 134 healthy controls aged 18 to 79 years. The corpus callosal white matter tracts were compared among patients with Imp-IA (*n* = 40), intact illness awareness (*n* = 60), and healthy controls. White matter disruption was measured with fractional anisotropy (FA) and mean diffusivity (MD). Group differences in FA were found in the splenium, with patients with Imp-IA having the lowest FA, which remained significant after controlling for sex, age, global cognition, and premorbid intelligence. No group differences in MD were observed. Splenial white matter tracts of the corpus callosum appear compromised in patients with Imp-IA. Transcallosal interhemispheric PPA white matter disruption may represent a “disconnection syndrome”, manifesting as Imp-IA in schizophrenia. Future studies are required to investigate the effects of noninvasive brain stimulation interventions, such as transcranial direct current or magnetic stimulation, on Imp-IA in association with white matter changes in patients with schizophrenia.

## Introduction

Impaired illness awareness or insight into illness (Imp-IA) occurs in ~ 50–80% of patients with schizophrenia.^1,2^ Imp-IA is a multidimensional construct consisting of impaired awareness of having a disorder, its symptoms, need for treatment, and negative consequences attributable to the disorder.^3,4^ Importantly, it is associated with treatment non-adherence and poor clinical outcomes in schizophrenia,^5,6^ which necessitates the need for an improved understanding of the pathophysiology of Imp-IA.

Although the neural mechanisms of Imp-IA in schizophrenia remain elusive, mounting evidence indicates that Imp-IA in schizophrenia may arise from interhemispheric imbalance, resulting in left hemisphere dominance, primarily within the posterior parietal area (PPA).^7–10^ This may represent or contribute to a transcallosal “disconnection syndrome”,^11–14^ that is, compromised white matter tract communications between the PPAs.^15,16^

Functional neuroimaging studies of Imp-IA provide evidence in support of the “dysconnection hypothesis”. The results from a functional magnetic resonance imaging (fMRI) study by our group assessing brain activity at the moment of illness denial during an illness awareness task revealed left hemisphere activations predominantly in the PPA in association with Imp-IA in schizophrenia.^8^ Similarly, in a resting state fMRI study, we showed that Imp-IA was associated with increased connectivity in the default mode network with left PPA (i.e., angular gyrus).^17^ Functional imaging studies by other groups have also collectively implicated the PPA and precuneus in association with Imp-IA.^9,18^ Taken together, the results of these imaging studies suggest that Imp-IA in schizophrenia may be a function of PPA dysfunction; however, the neuropathological correlates of this impairment are unclear.

Interventional studies provide further support for the “disconnection syndrome” in relation to PPA dysfunction. Although primarily designed to improve auditory hallucinations in schizophrenia, three recent studies found improvement in Imp-IA with the use of transcranial direct current stimulation to inhibit left temporoparietal area activity in patients with schizophrenia.^19–21^ Similar to our hypothesis, Chang et al.^20^ also speculate that the observed improvement in Imp-IA could be related to resolving the interhemispheric imbalance related to white matter disruption.^20^

Diffusion tensor imaging (DTI) is a technique that enables visualization of microstructural features of white matter fiber tracts that otherwise remain undetected using other MRI sequences.^15^ DTI commonly reports on indices of fractional anisotropy (FA) and mean diffusivity (MD).^22^ FA measures directionally dependent diffusion of water molecules, which is thought to assess white matter integrity that may reflect the density of axonal packing or damage to the axonal membrane, whereas MD is a measure of the magnitude of water diffusion along the axons, which provides an additional evaluation of white matter fiber density and organization.^22–24^ Although FA and MD are sensitive to microstructural changes, these measures are not specific to any etiology.^22^ Given that lower FA and higher MD values indicate disrupted white matter fiber tracts,^23^ DTI has been employed as a marker of neuropathology in a broad spectrum of diseases and neuropsychiatric disorders, such as schizophrenia.^23^

Numerous DTI studies in schizophrenia have used diffusion-based measures to reflect white matter abnormalities in several brain regions, including frontal, temporal and parietal areas.^24–27^ However, we are only aware of a few studies that have examined white matter integrity in relation to Imp-IA in schizophrenia.^28,29^ In patients with Imp-IA in schizophrenia, studies employing diffusion-based measures have reported deficits in a number of white matter tracts associated with cortical midline structures and in the splenium of the corpus callosum,^29^ as well as in various frontal, temporal, and parietal areas.^28^ Further, reductions in the density of oligodendrocytes and perineural oligodendrocytes (i.e., the cells responsible for making myelin sheaths), have been reported in parietal regions of patients with Imp-IA in schizophrenia.^16,30^ These studies collectively suggest abnormalities in brain white matter integrity in patients with Imp-IA in schizophrenia, which is suggestive of a “disconnection syndrome” within the white matter tracts linking implicated brain regions.

To test our conceptualization of Imp-IA in schizophrenia as a manifestation of a transcallosal interhemispheric “disconnection syndrome” between PPAs, we aimed to determine whether diffusion-based measures of white matter integrity were disrupted in the corpus callosal tracts linking the PPA (i.e,. splenium) in patients with Imp-IA in schizophrenia when compared with patients with intact illness awareness (Int-IA) and healthy controls (HC). We hypothesized that reduced FA values and increased MD values in the splenium of the corpus callosum (SCC) would be associated with Imp-IA in schizophrenia. For exploratory comparative purposes, we secondarily selected the other corpus callosal white matter tracts (i.e., genu (GCC), body (BCC), and whole corpus callosum (CC)); the other white matter tracts most commonly implicated in schizophrenia (i.e., bilateral uncinate fasciculus, internal capsule, and cingulum bundle)^31^; and whole-brain average FA and MD.

## Results

### Demographic and clinical characteristics

The demographic and clinical characteristics of participants are reported in Table 1. One hundred participants with schizophrenia and 134 healthy controls were included in the study. Of the 100 participants with schizophrenia, 40 were classified as having Imp-IA (Positive and Negative Syndrome Scale (PANSS) G12 mean score of 4.0 ± 1.2) and 60 were classified as having Int-IA PANSS G12 mean score of 1.2 ± 0.4). No group differences were found for age or sex. No patient group differences were found for age of illness onset, illness duration, antipsychotic drug chlorpromazine equivalents, and Abnormal Involuntary Movement Scale (AIMS) or Simpson-Angus Scale (SAS) scores. Education, Wechsler Test of Adult Reading (WTAR), and Mini-Mental State Examination (MMSE) scores were lowest in the Imp-IA group. The Imp-IA group had higher illness severity (i.e., PANSS total minus G12, PANSS positive, negative, and general psychopathology minus G12 subscales) when compared with the Int-IA group.

**Table 1:** Demographic and clinical characteristics of participants with schizophrenia and healthy controls

	Impaired illness awareness, Imp-IA (*n* = 40)		Intact illness awareness, Int-IA (*n* = 60)		Healthy Controls, HC (*n* = 134)		Statistic test (df)	*p* value
	Mean	SD	Mean	SD	Mean	SD		
Demographic								
Sex (%males)	63%		63%		49%		χ^2^(2) = 4.38; Chi-squared	0.112
Age	44.60	18.21	42.30	15.83	42.43	19.24	F(2, 231) = 0.24; ANOVA	0.784
Education (years)	12.43	2.65	13.77	2.40	15.33	1.83	F(2, 231) = 32.18; ANOVA	<0.0005^a^**
Cognitive								
WTAR (IQ)	105.65	15.55	113.81	11.72	117.66	7.78	F(2, 229) = 20.24; ANOVA	<0.0005^b^**
MMSE	28.30	1.88	29.10	1.36	29.34	0.92	F(2, 231) = 10.61; ANOVA	<0.0005^b^**
Illness severity								
Age of illness onset (years)	25.85	11.07	25.35	8.37	–		*t*(98) = 0.26*; t* test	0.798
Illness duration (years)	18.52	16.79	16.96	14.85	–		*t*(98) = 0.49; *t* test	0.628
Antipsychotic CPZ Equivalents	216.46	229.39	253.84	156.37	–		*t*(54) = − 0.85; t test	0.399
AIMS	1.48	3.92	0.52	1.33	–		*t*(45) = 1.49; *t* test	0.143
SAS	2.05	2.81	1.98	2.89	–		*t*(98) = 0.11; *t* test	0.909
PANSS G12	4.03	1.17	1.23	0.43	–		*t*(46) = 14.52; *t* test	<0.0005**
PANSS total minus G12	56.88	15.48	44.55	12.40	–		*t*(98) = 4.40; *t* test	<0.0005**
PANSS positive	17.20	6.74	11.50	4.29	–		*t*(60) = 4.75*; t* test	<0.0005**
PANSS negative	14.98	6.20	12.35	5.32	–		*t*(98) = 2.26*; t* test	0.026*
PANSS general minus G12	24.70	6.68	20.70	5.19	–		*t*(98) = 3.36*; t* test	0.001*

*ANOVA* Analysis of variance; *AIMS* Abnormal Involuntary Movement Scale; *CPZ* Chlorpromazine; *df* Degrees of freedom (between groups and within groups, where applicable); *MMSE* Mini-Mental State Examination; *PANSS* Positive and Negative Syndrome Scale; *SAS* Simpson-Angus Scale; *WTAR* Wechsler Test of Adult Reading

*p < 0.05

**p < 0.0005

^a^Post hoc Bonferroni significant group differences between Imp-IA & Int-IA, Imp-IA & HC, Int-IA & HC

^b^Post hoc Bonferroni significant group differences between Imp-IA & Int-IA, Imp-IA & HC

### Group differences in FA values

**Fig. 1:**
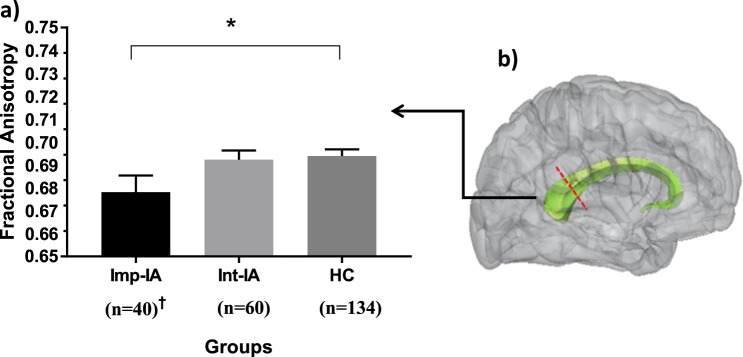
**a** Comparison of fractional anisotropy in the splenium of the corpus callosum among participants with schizophrenia and healthy controls. **b** Midsagittal view of the corpus callosum. The line (red) approximates the division between the body and splenium of the corpus callosum. *Imp-IA* impaired illness awareness; *Int-IA* intact illness awareness; *HC* Healthy controls. Error bars represent ±1 standard error. ^†^Significant difference between Imp-IA and HC after controlling for age, sex, education, premorbid intelligence, and global cognition (*p* = 0.041)

A mean group difference in FA values was found in the SCC (Table 2 and Fig. 1). Post hoc Bonferroni correction for multiple comparisons demonstrated a significant mean difference between the Imp-IA and HC groups (mean FA in Imp-IA = 0.678, mean FA in HC = 0.694, 95% CI = −0.31 to −0.00029, *p* = 0.044 (analysis of variance; ANOVA)). The result remained significant after controlling for age, sex, education, WTAR and MMSE scores (Mean FA difference between Imp-IA and HC = −0.016, 95% CI, −0.031 to −0.0005, *p* = 0.041 (ANCOVA)). No differences were found in other white matter tracts (F(32, 432) = 0.97, *p* = 0.518 (multivariate analysis of variance; MANOVA), Wilks’ Λ = 0.87, partial *η*^2^ = 0.067).

**Table 2:** Group differences in DTI measures of brain white matter tract integrity

	Imp-IA (*n* = 40)		Int-IA (*n* = 60)		HC (*n* = 134)		Statistic Test (df)	*p* value
	Mean	SD	Mean	SD	Mean	SD		
Average FA								
A priori region of interest								
Splenium (SCC)	0.678	0.046	0.692	0.031	0.694	0.034	F(2, 231) = 3.09; ANOVA	0.047*^a^
Other white matter tracts							F(32, 432) = 0.97; MANOVA	0.518
*Other corpus callosal tracts*								
BCC	0.572	0.056	0.589	0.040	0.591	0.048		
GCC	0.622	0.051	0.642	0.039	0.636	0.044		
Whole CC	0.618	0.049	0.635	0.033	0.635	0.039		
*Other white matter tracts previously associated with SCZ*								
ALIC_L	0.517	0.035	0.527	0.035	0.528	0.034		
ALIC_R	0.514	0.039	0.529	0.031	0.527	0.030		
CGC_L	0.510	0.054	0.517	0.036	0.519	0.042		
CGC_R	0.470	0.045	0.478	0.037	0.481	0.039		
CGH_L	0.435	0.046	0.451	0.055	0.442	0.047		
CGH_R	0.421	0.043	0.439	0.049	0.431	0.050		
PLIC_L	0.658	0.029	0.655	0.029	0.653	0.030		
PLIC_R	0.649	0.028	0.647	0.024	0.644	0.028		
RLIC_L	0.540	0.037	0.553	0.032	0.553	0.034		
RLIC_R	0.522	0.032	0.531	0.035	0.527	0.034		
UNC_L	0.474	0.058	0.487	0.052	0.492	0.058		
UNC_R	0.488	0.053	0.499	0.053	0.497	0.060		
Whole brain	0.379	0.024	0.386	0.019	0.386	0.020		
**Average MD**								
A priori region of interest								
*SCC*	0.00246	0.00020	0.00247	0.00018	0.00242	0.00020	F(2, 231) = 1.82; ANOVA	0.164
Other white matter tracts							F(32, 432) = 1.24; MANOVA	0.174
*Other corpus callosal tracts*								
BCC	0.00198	0.00037	0.00198	0.00028	0.00193	0.00033		
GCC	0.00202	0.00047	0.00195	0.00035	0.00188	0.00040		
Whole CC	0.00214	0.00033	0.00213	0.00025	0.00207	0.00029		
*Other white matter tracts previously associated with SCZ*								
ALIC_L	0.00176	0.00068	0.00169	0.00053	0.00165	0.00060		
ALIC_R	0.00170	0.00067	0.00156	0.00055	0.0015	0.00056		
CGC_L	0.00104	0.00013	0.00101	0.00008	0.00101	0.00011		
CGC_R	0.00101	0.00010	0.00098	0.00008	0.00098	0.00009		
CGH_L	0.00290	0.00026	0.00288	0.00034	0.00283	0.00030		
CGH_R	0.00274	0.00032	0.00282	0.00035	0.00267	0.00030		
PLIC_L	0.00098	0.00011	0.00097	0.00008	0.00096	0.00010		
PLIC_R	0.00093	0.00009	0.00092	0.00008	0.00091	0.00009		
RLIC_L	0.00149	0.00030	0.00143	0.00025	0.00141	0.00027		
RLIC_R	0.00150	0.00029	0.00146	0.00026	0.00142	0.00026		
UNC_L	0.00292	0.0003	0.00296	0.00029	0.00291	0.00031		
UNC_R	0.00278	0.00033	0.00284	0.00034	0.00271	0.00036		
Whole brain	0.00143	0.00019	0.00140	0.00014	0.00137	0.00016		

*ALIC* Anterior limb of internal capsule; *ANOVA* Analysis of variance; *BCC* Body of corpus callosum; *CGC* Cingulum (cingulate gyrus); *CGH* Cingulum (hippocampus); *CC* Corpus callosum; *df* Degrees of freedom (between groups, within groups); *FA* Fractional anisotropy; *GCC* Genu of corpus callosum; *HC* Healthy controls; *Imp-IA* Impaired illness awareness; *Int-IA* Intact illness awareness; L Left hemisphere; *MANOVA* Multivariate analysis of variance; *MD* Mean diffusivity; *PLIC* Posterior limb of internal capsule; *R* Right hemisphere; *RLIC* Retrolenticular part of internal capsule; *SCC* Splenium of corpus callosum; *SD* Standard deviation; *SCZ* Schizophrenia; *UNC* Uncinate fasciculus

*p < 0.05 after controlling for covariates and Bonferroni adjustments for multiple comparisons: age, sex, education, premorbid intelligence, and global cognition

^a^Post hoc Bonferroni significant group differences between Imp-IA & HC

### Group differences in MD values

No significant group differences were identified in the SCC and other white matter tracts (F(32, 432) = 1.24, *p* = 0.174 (MANOVA), Wilks’ Λ = 0.84, partial *η*^2^ = 0.084) (Table 2).

## Discussion

In this study, we aimed to investigate whether Imp-IA in schizophrenia across the adult lifespan was a manifestation of disrupted posterior corpus callosal tracts, representing a transcallosal interhemispheric “disconnection syndrome” between PPAs. As hypothesized, FA in the splenium was lowest in the Imp-IA group as compared to the Int-IA and HC groups. This result was specific to the splenium as there were no group differences in other white matter tracts, namely other corpus callosal regions (i.e., body or genu), tracts commonly implicated in schizophrenia (i.e., bilateral uncinate fasciculus, internal capsule, and cingulum bundle), or average whole-brain FA values. No group differences were found in the MD values. Taken together, our results seem to suggest that white matter disruption in the SCC, as measured by FA, is associated with Imp-IA in schizophrenia.

The results of our study are consistent with a recent investigation of white matter tract integrity in first-episode patients with schizophrenia aged 16 to 45 years, which found that Imp-IA was predicted by lower FA values in the SCC and cortical midline structures.^29^ By comparison, a voxel-wise DTI study investigating the association between Imp-IA and white matter integrity found compromised white matter in frontal, temporal, and parietal regions.^28^ While in another voxel-wise DTI study, increased FA was associated with Imp-IA in a small solitary region in the medial frontal gyrus.^32^ The inconsistency between the results of the latter two studies and our own may be attributable to methodological differences. These studies used a voxel-wise approach to explore the relationship between insight and white matter integrity, whereas our study employed a region-of-interest approach to investigate group differences. Further, these studies had relatively younger patient groups compared to our study.

Our finding of white matter compromise within the splenium advances the theory of interhemispheric imbalance between the PPAs contributing to left hemisphere dominance in patients with Imp-IA.^7,33,34^ In addition to their role in visuospatial reasoning and attention,^35^ the PPAs (i.e., temporoparietooccipital region, angular gyrus, and inferior parietal lobule) are important for other cognitive functions that might be linked to illness awareness.^34,36^ In one study, increased activation of the left PPA was observed during a self-reflection task in patients with schizophrenia.^9^ In the same study, illness awareness was also positively associated with PPA activation.^9^ Imp-IA may not be a simple function of structural or functional abnormalities within specific brain areas (e.g., left and right hemisphere PPAs), but could be related to disrupted communication (i.e., “disconnectivity”) between these key brain regions either representing or resulting in compromised white matter integrity.^34,37^

The relationship between left hemisphere dominance and interhemispheric disconnection in Imp-IA in schizophrenia may be explained by the “disconnection syndrome” put forth by Friston et al. (See Friston et al.^38^). According to this model, neuroanatomical compromise in white matter integrity may be a consequence of aberrant neuromodulatory (e.g., dopaminergic dysregulation) maintenance of the glutamatergic and gabaergic systems’ excitation–inhibition balance in schizophrenia. Plausibly, Imp-IA arises because the right PPA (as an integral component of a larger network) is unable to generate alternative perceptions of illness with enough salience to challenge the left hemisphere’s established self-perceptions of health. Alternatively, or concurrently, the left PPA network may actively suppress the right hemisphere’s attention to discordant perceptions of illness recognition and acceptance, resulting in Imp-IA. Through neuroplastic effects on synaptic function, persistent neuromodulatory dysregulation and accompanying false beliefs may ultimately result in white matter tract compromise.^32,38,39^ Thus, transcallosal interhemispheric disconnectivity between PPAs could either contribute to or represent the effects of persistent Imp-IA in schizophrenia.

Compromised white matter integrity in the splenium could represent a marker or therapeutic target to improve illness awareness and treatment adherence in patients with Imp-IA in schizophrenia.^8,40^ Evidence suggests that illness awareness in schizophrenia^19,20,41^ and other neuropsychiatric conditions (i.e., stroke)^42–44^ can be transiently improved with the use of noninvasive neurostimulation techniques, such as transcranial magnetic, direct current, and vestibular stimulation by enhancing interhemispheric function. In particular, three studies found marked improvements in Imp-IA in patients with schizophrenia after the application of fronto-temporoparietal transcranial direct current stimulation, which involved inhibitory cathodal stimulation of the left parietal area.^19–21^ Interestingly, the authors of one study attributed the improvement in Imp-IA to the restoration of interhemispheric balance.^20^ Serial neurostimulation may also facilitate splenial white matter tract integrity and lead to sustained improvements in illness awareness.^8,45^ However, future studies are required to determine whether multisession neurostimulation leads to sustained improvement in illness awareness in association with white matter changes in patients with schizophrenia.

Intriguingly, in the current study, no group difference in FA values was observed in the SCC between the Int-IA and HC groups, and between the Imp-IA and Int-IA groups. As such, it could be argued that the group difference found between the Imp-IA and HC groups is attributable to overall greater illness severity rather than simply Imp-IA. However, the significant result was specific to the splenium, the a priori white matter tract of interest. Moreover, group differences should have been observed in other callosal white matter tracts, other white matter tracts commonly associated with schizophrenia, or average whole-brain FA values (Table 2) if the difference was reflective of overall illness severity rather than Imp-IA. Alternatively, this result may indicate that treatment responsive patients have splenial white matter integrity comparable to HC participants. Some evidence suggests that antipsychotics, specifically clozapine, may improve white matter integrity in patients with schizophrenia.^46^ Notably, clozapine is the antipsychotic with the best evidence in treatment resistant schizophrenia^47^ and the only antipsychotic that appears to improve illness awareness independent of illness severity.^48^

Although there are few DTI studies that have used FA to measure white matter tract integrity in relation to Imp-IA in schizophrenia, we are not aware of any that have used MD. FA and MD both reflect white matter integrity (i.e., myelin or axonal membrane compromise, or axonal packing density), through directionality and magnitude of water diffusion, respectively.^22–24,49,50^ In our study sample, the extent of MD within the splenium or other white matter tracts was unrelated to Imp-IA.

This study has a few limitations. First, the effect of antipsychotics on white matter integrity is not well understood and may have contributed to the group differences in FA values.^46^ That being said, differences in antipsychotic drug chlorpromazine equivalents between Imp-IA and Int-IA groups did not reach statistical difference in our study. Second, although group differences in white matter tract integrity were observed after controlling for covariates (i.e., age, sex, education, MMSE scores, and premorbid IQ), other factors, such as illness severity appear to contribute to patient group differences in illness awareness or white matter tract structure. Third, MRI images were obtained using a 1.5T GE scanner, which could be a potential limitation for DTI fiber tracking given the availability of higher resolution scanners. Fourth, the influence of lateral ventricular enlargement present in schizophrenia on the SCC in the context of our findings is unclear and requires further exploration.^51,52^ Future studies exploring the corpus callosum should consider the possibilities of misregistration and partial volume effects due to ventricular enlargement in the region. Fifth, statistical analyses involving correlations between FA/MD values and demographic and clinical variables were limited by the use of the PANSS G12 item owing to its categorical design and limited response range. To overcome this, future studies should consider using more sophisticated measures of illness awareness, which may provide additional evidence to support the role of the corpus callosum in illness awareness in schizophrenia. Finally, although our total patient sample was larger in size than previous DTI studies, a post hoc power analysis revealed that our study was 41% powered to detect a difference between the two patient groups (i.e., Imp-IA and Int-IA) (Cohen’s *d* = 0.357). We expect that in order to achieve at least 80% power, future studies should increase the sample size of each group (*n* ≥ 100), which will help to detect differences in corpus callosal white matter integrity in relation to illness awareness.

In summary, we found that Imp-IA in schizophrenia was associated with lower FA specific to the splenium of the corpus callosum across the adult lifespan. This finding offers evidence in support of a posterior parietal interhemispheric “disconnection syndrome”, which may represent an underlying neuropathology of Imp-IA. Future interventional studies of the neuroplastic effects of antipsychotic drugs and brain stimulation should consider the enhancement of corpus callosal white matter integrity as an outcome. Improved white matter tract connectivity may, in turn, improve illness awareness, which could have a significant impact on treatment adherence and other clinical outcomes in patients with schizophrenia.

## Methods

### Participants

In this cross-sectional study, outpatients with schizophrenia (*n* = 100) and HC participants (*n* = 134) aged 18–79 years were recruited at the Centre for Addiction and Mental Health (CAMH) in Toronto, Canada, via advertisements, clinician referrals, and research registries. Written informed consent was obtained from each participant after full explanation of the study procedures and risks. All clinical assessments occurred at CAMH by a trained psychiatrist and DTI scans were obtained at the Toronto General Hospital in Toronto. Self-report medication histories were verified by the patients’ treating psychiatrists or by chart review. Urine toxicology screens were done as part of the initial assessment. Patients were included if they had a DSM-IV-TR diagnosis of schizophrenia. Exclusion criteria included: (i) serious, unstable medical illness or any concomitant major medical or neurological illness; (ii) acute suicidal and/or homicidal ideation; (iii) current substance abuse or any history of substance dependence; (iv) metal implants, cardiac pacemaker, claustrophobia or other limitations to participating in the MRI component of the study; and (v) head trauma resulting in a loss of consciousness for over 30 minutes. HC participants with first-degree relatives with a history of primary psychotic disorders were also excluded. The study was approved by the CAMH Research Ethics Board.

### Study assessments

PANSS^53^ was used to assess symptoms of schizophrenia. Illness awareness in patients with schizophrenia was evaluated using the PANSS “Lack of judgment and insight” (G12) item. This clinician-rated item is scored on a scale of 1 (Int-IA) to 7 (extremely Imp-IA). Patients were categorized into the Imp-IA group if their score was greater than or equal to 3 on the PANSS G12 item and into the Int-IA group if their score was <3. The cutoff value of 3 was chosen to ensure that the Int-IA group was only minimally contaminated by patients with impairment in illness awareness.^54,55^ WTAR^56^ was used to measure IQ. Cognitive function was assessed using the MMSE.^57^ Movement abnormalities and extrapyramidal symptoms were assessed using the AIMS^58^ and the SAS, respectively.^59^

### Diffusion tensor image acquisition, processing, and analysis

DTI images were acquired at Toronto General Hospital as part of a multimodal imaging protocol using an eight-channel head coil on a 1.5-T GE Echospeed (General Electric Medical Systems), which has maximum gradient amplitudes of 40 mT/m. A single-shot spin echo planar sequence with diffusion gradients in 23 noncollinear directions and *b* = 1000 s/mm^2^ was used, and two *b* = 0 images were obtained. Fifty-seven slices were obtained for whole-brain coverage oblique to the axial plane and parallel to the plane passing through the anterior and posterior commissures (i.e., anterior commissure-posterior commissure aligned). Slice thickness was 2.6 mm, and voxels were isotropic. The field of view was 330 mm, and the size of the acquisition matrix was 128 mm by 128 mm with an echo time of 85.5 ms and a repetition time of 15,000 ms. The entire sequence was repeated three times to improve the signal to noise ratio.

The three 23-direction repeats were first concatenated to each other. The concatenated files were corrected for eddy current induced distortions and head motion using FMRIB Software Library’s eddy_correct (FSL: http://fsl.fmrib.ox.ac.uk/fsl/fslwiki)^60^ and a brain mask was generated using T2-weighted b0 images through FSL-BET (Brain Extraction Tool). The pre-processed data were run through DTIFit to generate tensors and obtain FA and MD maps. These maps were processed using the ENIGMA-DTI pipeline (http://enigma.ini.usc.edu/ongoing/dti-working-group/).^61^ Tract-based spatial statistics was run on the maps to nonlinearly register all images to an ENIGMA-DTI FA template. All subjects’ maps were projected onto the ENIGMA-DTI skeleton that represents the middle of the tract of major white matter structures. The Johns Hopkins University DTI atlas in ICBM space (ICBM-DTI-81 white matter labels atlas)^62^ was applied to the 4D FA skeleton to obtain averaged FA for 46 fiber tracts. These steps were repeated to obtain MD for the same fiber tracts. The brain mask and vector directions of the skeletons were visually checked for quality control. Outlier scans with FA and MD values > 4 standard deviations of the mean values for all subjects in multiple tracts were excluded. Of the total 278 participants scanned, 44 were excluded from the present study due to the following reasons: scans could not be concatenated owing to different origins of DTI images (*n* = 2), outliers (*n* = 6), missing DTI scans/clinical data (*n* = 35), and duplication (*n* = 1).

For the purposes of the present study, the a priori tract of interest was the SCC. The other corpus callosal white matter tracts (i.e., GCC, BCC, and CC); other tracts commonly implicated in schizophrenia (i.e., anterior limb of internal capsule (ALIC), posterior limb of internal capsule (PLIC), retrolenticular part of internal capsule (RLIC), cingulum (cingulate gyrus) (CGC), cingulum (hippocampus) (CGH), uncinate fasciculus (UNC)); and whole-brain average were selected for exploratory comparative purposes.

### Statistical analysis

Statistical analyses of demographic, clinical, and neuroimaging variables were carried out using SPSS statistical software (Released 2016. IBM SPSS Statistics for Windows, Version 24.0. Armonk, NY: IBM Corp). Means and standard deviations were calculated for each variable for each of the three groups: Imp-IA, Int-IA and HC participants. ANOVA, covariance (ANCOVA), and two-tailed *t* tests were used for group analyses where appropriate. First, ANOVA and ANCOVA analyses were performed to assess group differences in the SCC. For ANCOVAs, the following covariates were included: age, sex, education, WTAR, and MMSE scores. Post hoc Bonferroni corrections for multiple testing were performed. Second, exploratory analyses in other white matter tracts (i.e., GCC, BCC, CC, ALIC, PLIC, RLIC, CGC, CGH, UNC) and for whole-brain FA and MD values were performed using tests of one-way MANOVA for comparative purposes. The significance level for tests was established at *p* ≤ 0.05.

### Reporting summary

Further information on research design is available in the Nature Research Reporting Summary linked to this article.

## Supplementary information

Life Sci Reporting Summary

## Data Availability

The data sets generated and/or analyzed during the current study are available from the corresponding author on reasonable request.

## Supplementary information

**Supplementary information** accompanies the paper on the *npj Schizophrenia* website (10.1038/s41537-019-0076-x).
